# Metal concentrations and biological effects from one of the largest mining disasters in the world (Brumadinho, Minas Gerais, Brazil)

**DOI:** 10.1038/s41598-020-62700-w

**Published:** 2020-04-03

**Authors:** Cristiane dos Santos Vergilio, Diego Lacerda, Braulio Cherene Vaz de Oliveira, Echily Sartori, Gabriela Munis Campos, Anna Luiza de Souza Pereira, Diego Borges de Aguiar, Tatiana da Silva Souza, Marcelo Gomes de Almeida, Fabiano Thompson, Carlos Eduardo de Rezende

**Affiliations:** 1 Laboratório de Ecotoxicologia, Departamento de Biologia, Centro de Ciências Exatas Naturais e da Saúde, Universidade Federal do Espírito Santo - Campus Alegre. Alto Universitário, S/N, Guararema, Alegre, Espírito Santo 29.500-000 Brasil; 2 Laboratório de Ciências Ambientais, Centro de Biociências e Biotecnologia, Universidade Estadual do Norte Fluminense Darcy Ribeiro. Avenida Alberto Lamego, 2000, Parque Califórnia, Campos dos Goytacazes, Rio de Janeiro, 28013-602 Brasil; 3 Laboratório de Microbiologia, Centro de Ciências da Saúde, Instituto de Biologia, Universidade Federal do Rio de Janeiro, Ilha do Fundão, anexo ao bloco A, Rio de Janeiro, 219.449-70 Brasil

**Keywords:** Biogeochemistry, Environmental sciences

## Abstract

The rupture of the Brumadinho mining tailings dam in Brazil is considered one of the largest mining disasters in the world, resulting in 244 deaths and 26 missing people, in addition to the environmental consequences. The present study aims to evaluate the concentrations of multiple elements and the biological effects on water and sediments of the Paraopeba River after the Brumadinho Dam rupture. The tailings are formed by fine particulate material with large amounts of Fe, Al, Mn, Ti, rare earth metals and toxic metals. In the water, the levels of Fe, Al, Mn, Zn, Cu, Pb, Cd and U were higher than those allowed by Brazilian legislation. In the sediments, Cr, Ni, Cu and Cd levels were higher than the established sediment quality guidelines (TEL-NOAA). The differences in metal concentrations in the water and sediments between the upstream and downstream sides of the dam illustrate the effect of the tailings in the Paraopeba River. Toxicological tests demonstrated that the water and sediments were toxic to different trophic levels, from algae to microcrustaceans and fish. The fish exposed to water and sediments containing mine ore also accumulated metals in muscle tissue. This evaluation emphasizes the necessity of long-term monitoring in the affected area.

## Introduction

In less than four years, two major environmental tragedies involving mining dams occurred in Brazil. The first incident is considered the industrial disaster with the greatest environmental impacts in Brazilian history and the largest of the world involving tailings dams. The rupture of the Fundão Dam in the subdistrict of Bento Rodrigues, 35 km from the municipality of Mariana in Minas Gerais State on November 5, 2015, resulted in 19 deaths due to the release of more than 40 million m^3^ of tailings that were transported to the mouth of the Doce River^1^. The 668 km length of affected water by Fundão tailings is the largest ever recorded^2^. The second incident occurred on January 25, 2019, when a tailings dam (“Dam B1”) failed at Córrego do Feijão mine in the city of Brumadinho, also in Minas Gerais State, releasing approximately 12 million m^3^, which directly affected the administrative area of the company and parts of the nearby communities^3^, resulting in 244 deaths and 26 missing people, as some bodies were completely buried in the mud and never found^4^.

**Figure 1:**
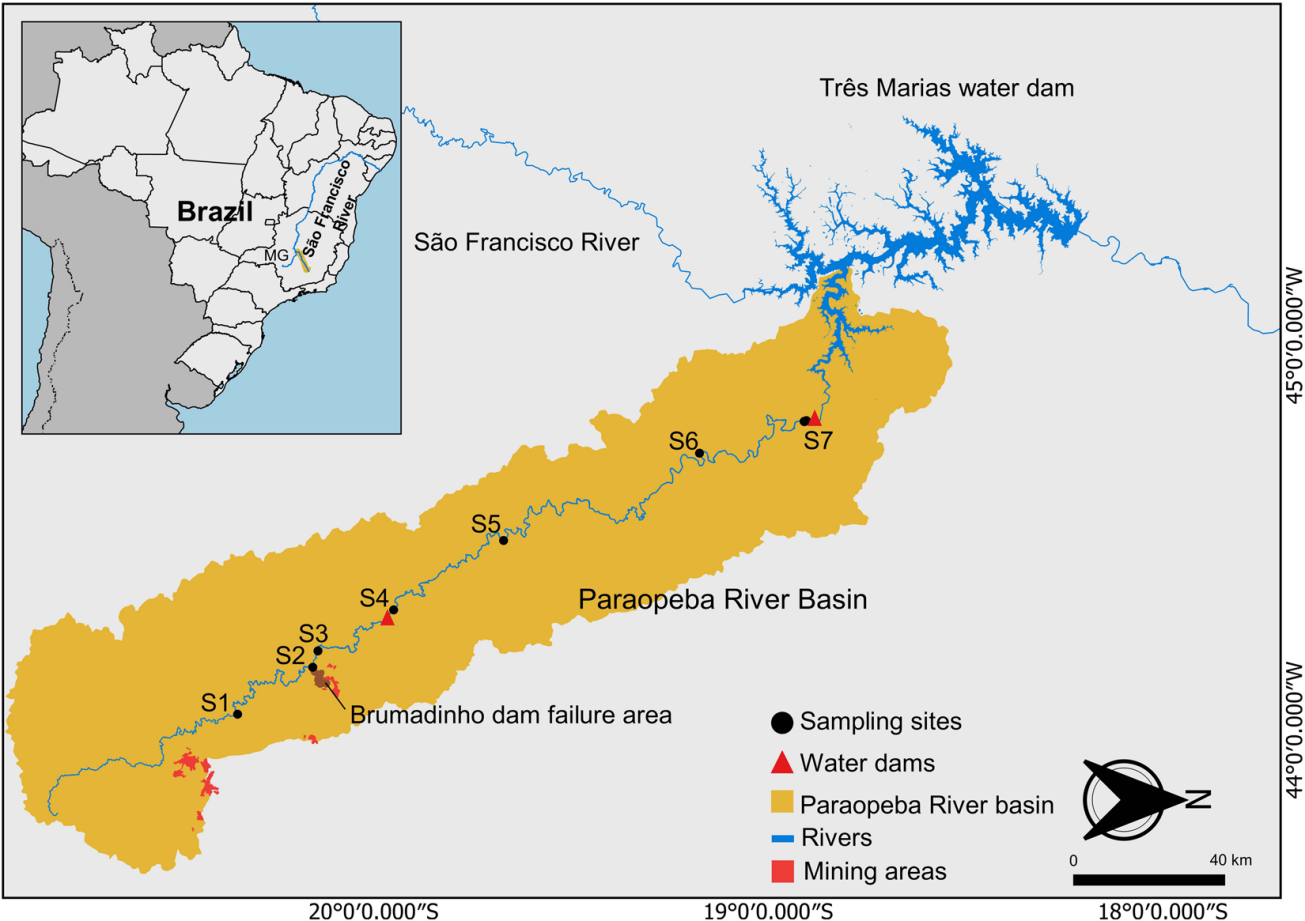
Map of the sampling sites with the trajectory of the mining tailings along the Paraopeba River. S1 - Moeda is upstream of the dam, S2 - Tailing is the site where a representative tailing sample was collected. S3 – Brumadinho, S4 – Juatuba, S5 – São José da Varginha, S6 – Angueretá, S7- Retiro Baixo are dowstream of the tailings dam. Data source: IBGE, 2019^32^.

Although the previous evaluation by the Brazilian Mining Agency classified Dam B1 in Brumadinho as “low risk of collapse and high potential of associated damage”^5^, the rupture of the Brumadinho Dam led to the release of iron ore tailings into the Feijão stream that is a tributary of the Paraopeba River^6^ (Supplementary Video, Fig. 1). Along the way, the volume of tailings led to an immediate increase in water turbidity (Supplementary Fig. S1a), impacts on the water supply in the municipalities provided by the Paraopeba River, and damage to fauna and flora, tourism, and especially to the population living in the affected areas^6^ (Supplementary Fig. S1b). The human, social and environmental impacts of this catastrophe are immeasurable.

After initial dam disruption, much of the suspended particulate matter present in the water column followed its course along the riverbed, was deposited and consolidated in the bottom sediments, and was absorbed by organisms as part of the aquatic food chain^2,7^. Due to the iron ore source, which is rich in diverse trace elements, the prolonged exposure of organisms can cause acute and long-term toxicological effects^8^. In particular, some trace elements, such as Ba, Pb, As, Sr, Fe, Mn and Al, have high mobility from the tailings to water^9^ with the potential to induce cytotoxicity and DNA damage, as already demonstrated for Fundão tailings^8,9^.

Therefore, monitoring of metal levels is necessary along the course of the released mining waste. Beyond the chemical analysis in abiotic and biotic matrices, further studies to evaluate the toxicological consequences for different species present in the water column, from primary producers to higher levels, consider the overall environmental and human risk of contamination. This evaluation, based on an assessment of the biological, chemical and physical properties of the system, contributes to better water resource protection and conservation and helps water managers plan rehabilitation^10^.

Toxicological tests using different indicator organisms have been implemented in several countries as requirements of environmental agencies in the licensing and inspection of potentially polluting activities, as well as in the monitoring of water quality^11^. From this perspective, the present study aims to evaluate the concentrations of multiple elements and the biological effects on water and sediments of the Paraopeba River after the mining tailings dam rupture in Brumadinho in southeast Brazil.

## Results

### Characteristics of the tailings

The tailings released from the ruptured Dam B1 at Córrego do Feijão mine are fine particulate material (30.3% sand and 69.7% silt-clay) composed mainly of Fe (264.9 mg/g), Al (10.8 mg/g), Mn (4.78 mg/g) and Ti (0,43 mg/g), among other elements. Trace toxic metals are also present, such as U (1457.4 µg/g), Cd (30.94 µg/g), Pb (14.64 µg/g), As (4.69 µg/g), Sn (547.4 µg/g), and Hg (101.3 ng/g), and rare earth metals, such as In (210.2 µg/g) and Ga (92.34 µg/g), among others (Supplementary Table S5).

### Consequences of the tailings dam along the Paraopeba River

**Figure 2:**
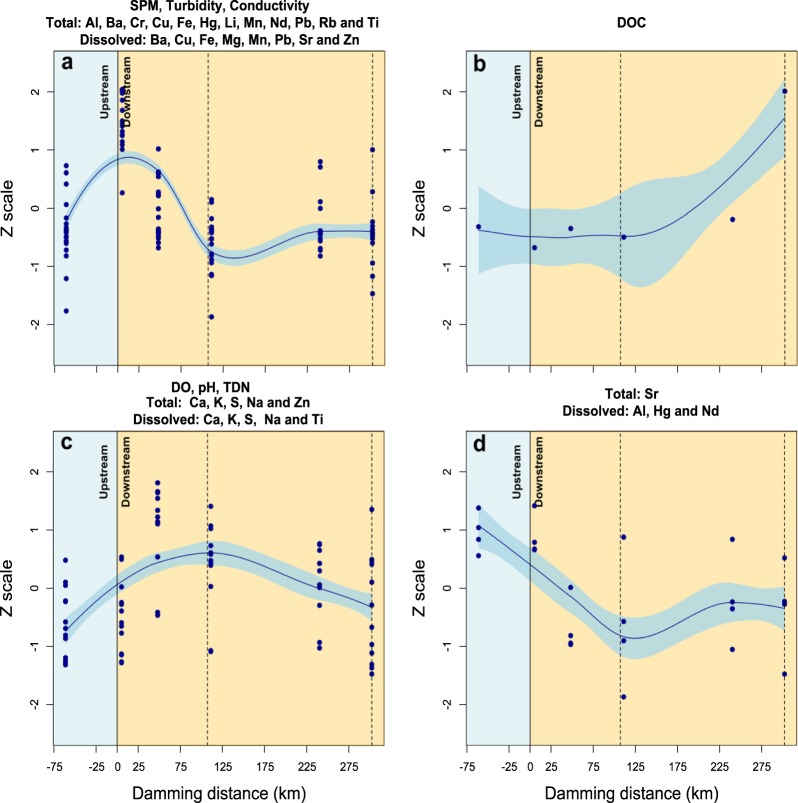
Association between the water variables of the Paraopeba River five days after the Brumadinho mine tailings dam rupture. The values of the observations were transformed to a z-scale to make the variables comparable. (**a**) The first group of variables clustered by the same behavior formed by SPM, turbidity, conductivity, and total Al, Ba, Cr, Cu, Fe, Hg, Li, Mn, Nd, Pb, Rb and Ti concentrations and dissolved Ba, Cu, Fe, Mg, Mn, Pb, Sr, and Zn concentrations. (**b**) The second group formed by DOC. (**c**) The third group formed by pH, DO, TDN, total Ca, K, S, Na and Zn concentrations and dissolved Ca, K, S, Na and Ti concentrations. (**d**) The forth group formed by the total Sr and dissolved Al, Hg and Nd concentrations. The light blue shading identifies the standard error of the regression model. The dashed lines represent two water dams along the river. SPM: Suspended Particulate Material, DOC: Dissolved organic carbon, DO: Dissolved oxygen, TDN: Total Dissolved Nitrogen.

The tailings dam rupture induced an immediate increase in water turbidity, reaching values of 3000 NTU closest to the failure area (Brumadinho – 5.2 km). The increase in turbidity levels was accomplished by the high amount of superficial particulate material in the water column (516 mg/L in Brumadinho – 5.2 km) (Fig. 2a, Supplementary Table S2).

### Total and dissolved elements in the water

In the water, the analyzed parameters were arranged in four groups (Fig. 2a–d). The first group included the most variables (SPM, turbidity, conductivity, and total Al, Ba, Cr, Cu, Fe, Hg, Li, Mn, Nd, Pb, Rb and Ti concentrations and dissolved Ba, Cu, Fe, Mg, Mn, Pb, Sr, and Zn concentrations) that showed lower levels upstream of the rupture area (S1 – Moeda: 61.3 km), reached a peak in Brumadinho (S2: 5.2 km) and decreased their levels with increasing distance from the dam (Fig. 2a). The DOC showed a distinct behavior with a tendency of increased levels only at the last sampling site (S7 – Retiro Baixo: 302 km downstream from the rupture area) (Fig. 2b). The pH, DO, TDN, total Ca, K, S, Na and Zn concentrations and dissolved Ca, K, S, Na and Ti concentrations showed peak levels 111 km downstream from the rupture area at S5 - São José da Varginha; however, the lowest levels of these parameters were observed both upstream and downstream of the rupture area (Fig. 2c). The total Sr and dissolved Al, Hg and Nd concentrations were grouped due to high levels in the upstream part of the rupture area, with a tendency to decrease as far as S5 (São José da Varginha – 111 km), with minor alterations in the following sampling sites (Fig. 2d).

Some elements (Al, Cd, Fe, Hg, Mn, P and V) and rare earth metals (In and Gd) increased in concentration up to 10 times (mean = 17 times) below the dam rupture area (S2 – Brumadinho – 5.2 km) in relation to upstream levels (Supplementary Table S4). In particular, Cd, Mn, P and In levels increased by at least 70 times.

In addition, the concentrations of total Cd, Mn, Pb, Zn and U and dissolved Al and Fe presented higher levels than those allowed by the Brazilian National Council of the Environment - CONAMA for class I waters (human supply after simplified treatment); in particular, Al, Fe, Mn and U showed inclusive levels above the maximum allowed for class III water (human supply after conventional or advanced treatment), raising concern about the possible effects on biota and human health (Supplementary Table S4).

### Elements in the sediments

**Figure 3:**
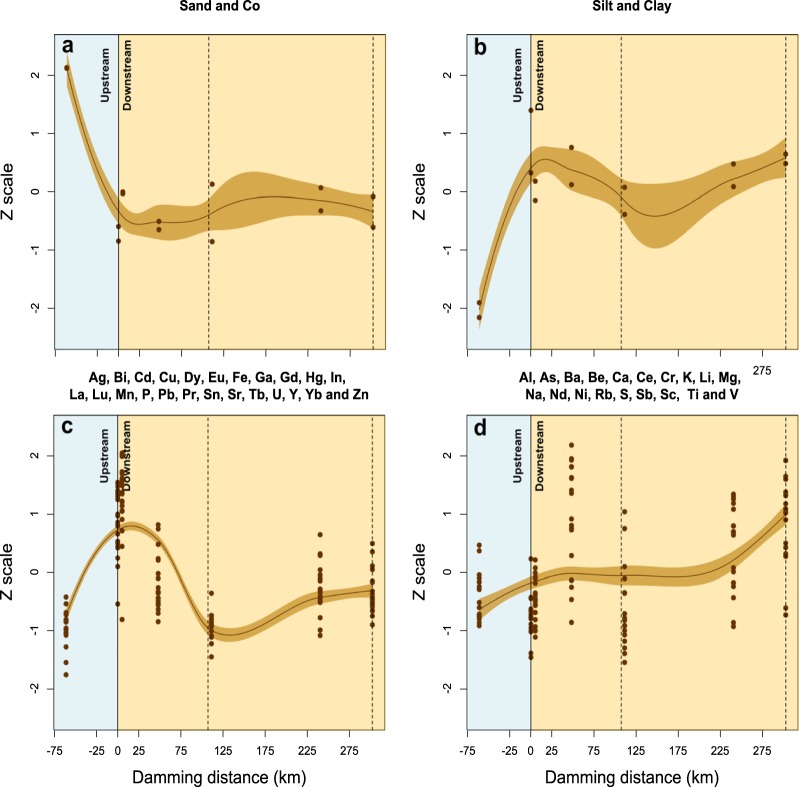
Association between the superficial sediment variables of the Paraopeba River five days after the Brumadinho mine tailings dam rupture. The values of the observations were transformed to a z-scale to make the variables comparable. (**a**) The first group of variables clustered by the same behavior formed by Co and sand fraction. (**b**) The second group formed by silt and clay fractions. (**c**) The third group formed by Ag, Bi, Cd, Cu, Dy, Eu, Fe, Ga, Gd, Hg, In, La, Lu, Mn, P, Pb, Sn, Sr, Tb, U, Pr, Y, Yb and Zn. (**d**) The forth group formed by Al, As, Ba, Be, Ca, Ce, Cr, K, Li, Mg, Na, Nd, Ni, Rb, S, Sb, Sc, Ti and V. The brown shading identifies the standard error of the regression model. The dashed lines represent two water dams along the river.

The parameters determined in the sediments were arranged in four groups (Fig. 3a–d). The first group was formed by the element Co and its association with the sand fraction (Fig. 3a), which showed decreases below the dam rupture area and did not fluctuate with increasing distance from the dam. Conversely, the second group composed of silt and clay fractions (Fig. 3b) increased their levels downstream from the dam. The third group consisted of the elements Ag, Bi, Cd, Cu, Dy, Eu, Fe, Ga, Gd, Hg, In, La, Lu, Mn, P, Pb, Sn, Sr, Tb, U, Pr, Y, Yb and Zn (Fig. 3c) and showed peak levels in Brumadinho (S3–5.2 km), immediately below the dam rupture area, followed by decreases in concentrations after S4. The last group of elements (Al, As, Ba, Be, Ca, Ce, Cr, K, Li, Mg, Na, Nd, Ni, Rb, S, Sb, Sc, Ti, and V) showed less oscillation in their levels between the upstream and downstream sides of the dam (Fig. 3d).

The increases in the concentrations of these elements in the sediments are a matter of great concern, especially considering that some of them, such as Cr, Ni, Cu, Cd, Hg and As, presented concentrations higher than the TEL and may cause adverse effects to organisms (Supplementary Table S5). Cd levels in certain areas were even above the PEL for almost all sampling sites downstream of the dam (Supplementary Table S5).

### Enrichment in metal levels along the Paraopeba River

**Figure 4:**
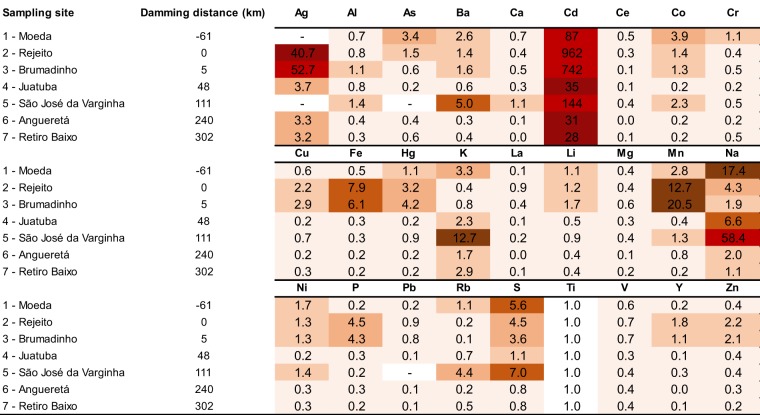
Enrichment factors (EFs) from the tailings and sediments of sampling sites along the Paraopeba River. The shades of brown indicate the following: 1 indicates no enrichment; <3 is minor enrichment; 3–5 is moderate enrichment; 5–10 is moderately severe enrichment; 10–25 is severe enrichment; 25–50 is very severe enrichment; and >50 is extremely severe enrichment.

The elements Ag, Cd, Cu, Fe, Hg, La, Li, Mn, P, Pb, Y and Zn showed higher enrichment factors in both the tailings and Brumadinho (S3–5.2 km), the first sampling site downstream from the dam area (Fig. 4). Meanwhile, As, Cd, Co, K, Na and S showed high enrichment levels since  Moeda (−61.3 km), upstream of the dam rupture area (Fig. 4). In both cases, there is a tendency of lower enrichment farther from the Dam, with the exception of Ba, Cd, K, Na, Rb and S, which showed at least moderate enrichments along the Paraopeba River (Fig. 4).

### Biological effects of the tailings dam rupture

**Figure 5:**
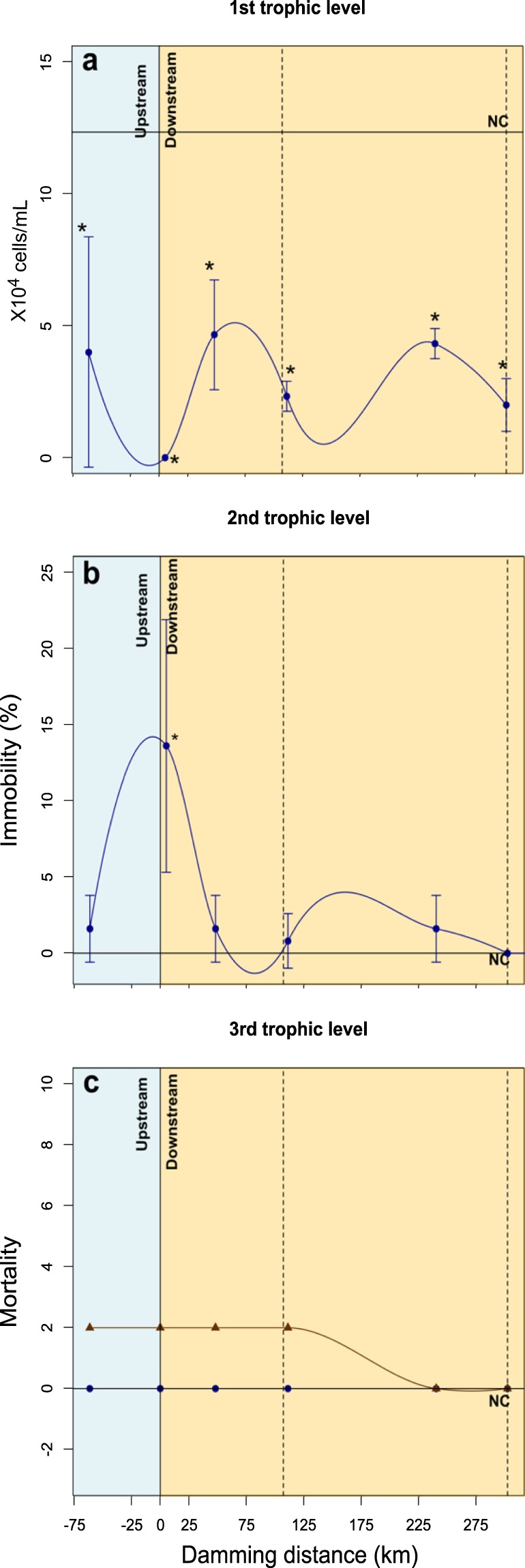
Toxicological tests with representative species of different trophic levels exposed to the water (blue) or sediment (brown) of Paraopeba River five days after of the Brumadinho dam rupture: (**a**) chlorophycea algae - *Raphidocelis subcaptata* (primary producer), (**b**) microcrustacean - *Daphnia similis* (primary consumer), and (**c**) fish - *Danio rerio* (secondary consumer). The dashed lines represent two water dams along the river. *significant compared to negative control.

Toxicological tests demonstrated that the water and sediments with mine ore have the potential to induce toxic effects at different trophic levels, from primary producers such as algae to primary and secondary consumers such as microcrustaceans and fish species (Fig. 5). The chlorophyceae algae *Raphidocelis subcapitata*, indicative of the first trophic level (primary producers) was more sensitive in environmental monitoring, demonstrating inhibition in cell growth after exposure to water from all sampled sites (Fig. 5a). The microcrustacean *Daphnia similis*, indicative of the second trophic level (primary consumers), showed immobility after exposure to Brumadinho (S3) water, the sampling site immediate downstream from the dam rupture (Fig. 5b). The *Danio rerio* fish, indicative of the third trophic level (secondary consumers), showed no mortality after water exposure; however, the induction of 20% mortality occurred in fish exposed to S1 – Moeda, S3 – Brumadinho, S4 – Juatuba and S5 – São José da Varginha sediments (Fig. 5c).

### Fish accumulation after exposure to the Paraopeba River water and sediments

**Figure 6:**
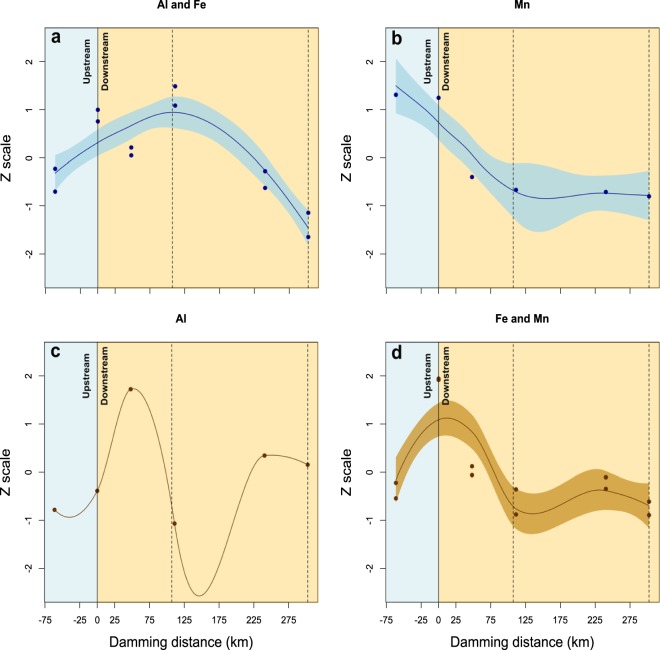
Association between the elements in the muscle of *D. rerio* fishes exposed to the water (blue) or sediment (brown) of the Paraopeba River five after days of the Brumadinho mine tailings dam rupture. The values of the observations were transformed to a z-scale to make the variables comparable. (**a**) Al and Fe in muscle of the fishes exposed to the water. (**b**) Mn in muscle of the fishes exposed to the water. (**c**) Al in muscle of the fishes exposed to the sediment. (**d**) Fe and Mn in muscle of the fishes exposed to the sediment. The light blue shading identifies the standard error of the regression model. The dashed lines represent two water dams along the river.

Due to the main composition of the tailings with Fe, Al and Mn, the potential for fish accumulation of these metals was evaluated (Supplementary Table S6). The fish exposed to water or sediments showed the same order of metal accumulation in muscle tissue, i.e., Fe > Al > Mn (Supplementary Table S6). The muscle accumulation changed with the exposure matrix (Fig. 6). When the fish were exposed to water, the peaks of Al and Fe accumulation were observed after exposure at S5 (São José da Varginha – 111 km) (Fig. 6a), while for Mn, the accumulation was higher after exposure at the sampling points closest to the dam (S1 - Moeda: − 61.3 km and S3 – Brumadinho: 5.2 km) (Fig. 6b). When the fish were exposed to the sediments, the Al accumulation showed a behavior distinct from those of Fe and Mn. However, the peak of muscle accumulation in both cases was observed after exposure to Brumadinho (S3–5.2 km) sediments (Fig. 6c,d).

## Discussion

Two environmental disasters of large proportions involving mining dams occurred in Brazil in less than four years. Iron ore tailings released by the ruptures of both the Fundão and Brumadinho Dams have similar features, such as fine particulate material, with a majority of silt-clay particles and a predominance of Al, Fe, and Mn^9,12,13^. The major components of the Brumadinho tailings were also observed by the Brazilian Geological Service^14^. The similarity of the tailings released by the Brumadinho and Fundão Dams occurred because both mine complexes are located in the Quadrilátero Ferrífero region in southeast Brazil, which is rich in iron deposits^15^.

### Immediate consequences of the tailings dam rupture along the Paraopeba River

The turbidity levels reached 3000 NTU and achieved the highest levels of SPM (516.14 mg/L) at the sampling site immediate from the dam rupture (Brumadinho – 5.2 km from the rupture area). According to the Brazilian Geological Service, the turbidity levels in the Paraopeba River reached even higher values from 11 to 22.000 NTU after the dam rupture^6^, with values above 1.100 NTU and incompatible with the historical series from 2002 to 2018^6^ and with the limit of Brazilian legislation (100 NTU) for class 2 and 3 waters (for human supply after conventional or advanced treatment)^16^. For comparison, after the Fundão Dam rupture, the turbidity reached the order of hundreds of thousands of NTU at the Doce River^2,17^, with SPM reaching 33.000 mg/L^7^ in the first days of monitoring, closest to the break site.

After the Brumadinho (S3) sampling site, the turbidity and SPM levels decreased to historic values. This decrease was observed because the sampling campaign occurred only 5 days after the rupture, and the dense released material moved very little within the Paraopeba River. These results are in accordance with the information regarding the plume movement along the Paraopeba River^18,19^. The estimated velocity of the plume was approximately 0.55 km/h on the sampling day, and the visual aspect of the intense color of the tailings was observed in the municipality of Pará de Minas (42 km)^18,19^. The lowest turbidity values at Juatuba (S4–48 km downstream from the rupture area) indicated that this sampling site was still not affected by the mud. In fact, the conservation of other physical and chemical parameters, such as SPM, conductivity, pH, OD and temperature, from Juatuba (48 km) to Retiro Baixo (S7) also indicated that these sampling sites were not yet affected by the tailings dam rupture (Supplementary Table S2).

### Total and dissolved elements in the water

In relation to the metal concentrations in the water, the dissolved Al, Ba, Fe and Pb levels (Al: 5×, Ba: 5×, Fe: 2x and Pb: 4×) were higher than the historic means for the Paraopeba basin^20^. According to CPRM^20^, only the dissolved Al and Fe presented levels above the legal limit established by Brazilian law (CONAMA 357/2005) in a sampling that occurred during the same period. However, the present results indicated that the concentrations of total Cd, Mn, Pb, Zn, and U and dissolved Al and Fe presented values higher than the levels allowed by Brazilian law for class I water (for human supply after simplified treatment), raising concern about the possible effects on biota and human health.

These differences may be due to methodological issues. In the present study, the Al, Fe and Cu concentrations were compared to the dissolved fraction in the Brazilian legislation, while the total levels were used for the other elements, according to the recommendations of Brazilian and international protocols. The analysis of all elements performed by the Brazilian Geological Service in the water samples involved a filtration process, and the results were representative of the dissolved fraction of metals in the water.

According to the historical series between 2000 and 2018, the incidence of levels above those in Brazilian law (CONAMA 357/2005) was already demonstrated along the Paraopeba basin for dissolved Fe (35%), total Mn (90% upstream and 50% downstream from the dam rupture area), dissolved Al (30%), Pb (10%), As, Cr, Ni, Zn, dissolved Cu and Cd (less than 5%)^21^. These variations may be natural due to the high background contents of the elements in rocks and soils or due to iron and gold deposits, together with the pollution caused by intense human activity in the region^15^. However, the potential impact of tailings dam rupture can worsen this situation and cannot be ignored because some elements, such as Al, Ba, Cd, Ce, Co, Er, Fe, Gd, Hg, In, La, Li, Lu, Mn, Ni, P, Pb, Sc, Ti, U, V, Y, and Zn, increased their levels in the water downstream of the dam rupture area in relation to upstream levels as a direct influence of the tailings passage (Supplementary Table S4).

Most of the water parameters analyzed (SPM, turbidity, conductivity, and total Al, Ba, Cr, Cu, Fe, Hg, Li, Mn, Nd, Pb, Rb and Ti concentrations and dissolved Ba, Cu, Fe, Mg, Mn, Pb, Sr, and Zn concentrations) presented concentration peaks at the Brumadinho sampling site (S3–5.2 km), followed by decreases in their levels with increasing distance from the dam (Fig. 2a). This result occurred because during the sampling period, the plume had extended 42 km^18,19^. Therefore, the effect of the tailings on the increases in metal concentrations in Juatuba water (S4–48 km) was not as evident at this moment. Beyond these facts, the Dam B1 rupture led to the deposition of tailings at the confluence, partially blocking the Paraopeba River^6^, which might shave lowed the velocity of mud transport along the course and consequently affected the metal concentrations in the water. The DOC (Fig. 2b), pH, DO, TDN, total Ca, K, S, Na and Zn and dissolved Ca, K, S, Na and Ti (Fig. 2c) showed less influence associated with the dam rupture.

The higher levels of total Sr and dissolved Al, Hg and Nd (Fig. 2d) upstream of the dam rupture area might reflect the impacts that already existed in the region, such as those from other mining areas (see the mining areas in Fig. 1), plantations, livestock, and urbanization, which influence concentrations for certain elements^20^. Other parameters, such as SPM, turbidity, conductivity, and total Al, Ba, Cr, Cu, Fe, Hg, Li, Mn, Nd, Pb, Rb and Ti concentrations and dissolved Ba, Cu, Fe, Mg, Mn, Pb, Sr, and Zn concentrations, also showed elevated levels upstream from the dam rupture area. Higher levels upstream of the dam (-10 km) had already been observed for some elements, especially dissolved Fe and total Mn^21^. Previous data from the region show a deeply impacted basin with high levels of Hg, Mn, Fe and Al in the soil, sediments and water^20^. However, the mud release along the Paraopeba River accentuated these problems along the basin.

### Elements in the sediments

Many elements (Ag, Bi, Cd, Cu, Dy, Eu, Fe, Ga, Gd, Hg, In, La, Lu, Mn, P, Pb, Sn, Sr, Tb, U, Pr, Y, Yb and Zn) showed peak levels at Brumadinho (S3–5.2 km), the sampling site immediately downstream from the dam rupture area, followed by decreases in concentrations beyond S4. The behavior of these elements indicates that the mud from Dam B1 still had not reached Juatuba (S4–48 km). The same pattern of higher Fe, Mn, Pb, Hg, Cu, Ni, and Zn levels in the sediments after the dam rupture (between distances of 20 and 42 km from the dam) followed by decreases between 59 and 318 km has been observed by other monitoring programs^22^. The present data corroborate the information that during the sampling period, the mud traveled approximately 42 km along the Paraopeba River^18,19^.

Another group of metals in the sediments (Al, As, Ba, Be, Ca, Ce, Cr, K, Li, Mg, Na, Nd, Ni, Rb, S, Sb, Sc, Ti, and V) showed less oscillation in their levels between the upstream and downstream sides of the dam. These metals showed less influence from the passage of tailings and might reflect high natural levels from rocks and soils with deposits of iron and gold and the presence of other mining areas (see Fig. 1), plantations, livestock and urbanization along the basin that contribute to the metal concentrations in the sediments.

The present study and other data from the same period showed As, Cu, Cr, Hg, Pb, and Ni levels above the TEL. Our data also show Cd levels even above the PEL for almost all sampling sites downstream of the dam^22^. These levels emphasize the possibility of inducing adverse effects in organisms because the elements deposited in the bottom sediments can eventually be remobilized to the water column, available for biotic accumulation and incorporation of metals in the trophic chain. Cu and Cr values above the levels set by Brazilian legislation for drainage sediment (CONAMA 454/2012 resolution) were also observed in Brazilian Geological Service monitoring^20^.

### Enrichment factor

The correlations made with the elements present in the tailings and those present in the sediments are important to determine one or a set of marker elements for the presence of the tailings. The enrichments observed for Ag, Cd, Cu, Fe, Hg, La, Li, Mn, P, Pb, Y and Zn might be used as indicators of mud presence along the Paraopeba River after the dam rupture because higher values were observed only in the tailings and at Brumadinho (S3–5.2 km), immediately downstream of the dam area (Fig. 4). Ag, Fe, La, Mn, P, and Y also showed enrichments in comparison with 2011 levels^20^. Meanwhile, As, Cd, Co, K, Na and S showed high enrichment levels since upstream the dam rupture area, which might be used to show the influence of natural and/or anthropogenic activities in the upper part of the basin.

In particular, for Al, despite the enrichment observed downstream from the dam rupture, this element is not a good marker for the observation of the movement of tailings along the Paraopeba River. Al in the sediments was observed throughout the river, even in sections that had not yet been impacted by the tailings in both the present study and data from other monitoring programs during the same sampling period^22^.

### Biological effects of the tailings dam rupture

Including various organisms in toxicity evaluations ensures a better integrated response because the test organisms show different sensitivities to the contaminants, which in turn maximizes the chance to detect a response after exposure to a sample with unknown chemical composition^10^. The toxicological tests demonstrated that the water and sediments with mine ores from the Brumadinho Dam rupture were toxic to different trophic levels. The unicellular algae were more sensitive because growth inhibition was observed at all sampled sites, especially beyond Brumadinho (S3), where water rich in tailings induced complete inhibition of algae growth. The oscillations in algal growth after the dam failure was a likely result of both eutrophication and the existence of secondary pollution in a deeply impacted region^23^, with large areas of mining, plantations, livestock and the proximity of urban centers^20^. The incidences of immobilization in microcrustaceans and fish deaths also demonstrate the toxicity of the Paraopeba River water and sediments. These data strongly support the susceptibility of the natural biota in the Paraopeba River and reinforce the need for long-term monitoring, considering not only the metal levels in abiotic matrices but also the biological effects in the local trophic chain, through toxicological evaluations and field studies. The sediments showed a higher toxic potential than the water due to the 20% mortality occurrence in the fish exposed to S1 (Moeda: − 61.3 km) to S5 (São José da Varginha: 111 km) sediment samples.

### Accumulation of metals in fish

The fish exposed to water and sediments containing mine ore from the Brumadinho Dam rupture accumulated metals in their muscle tissue. The metal accumulation in the muscular tissue of fish exposed to water and sediments shows that these elements are available for accumulation in the biota, suggesting a possible incorporation into the trophic chain and eventual risk of human contamination through the consumption of contaminated fish. The metal accumulation in fish can be related to the oscillation of metal levels in the water and sediments between the sampling sites, which demonstrates the importance of biomonitoring considering the whole path of the tailings along the Paraopeba River.

### Future consequences of the tailings dam rupture along the Paraopeba River

The tailings composition with large amounts of Fe, Al, Mn, and Ti, together with the high concentrations of toxic metals such as U, Cd, Pb, As and Hg and rare earth metals such as In and Ga, among others, has aroused great concern. The Paraopeba River is responsible for at least 4% of the water flow and at least 11% of the suspended sediment flow to the São Francisco River^21^. The initial data from the present study and the official governmental information indicate no evidence that the mining tailings from Dam B1 exceeded the limits of the Retiro Baixo reservoir (more than 302 km from the dam) and the São Francisco River^24^. However, with time, much of the SPM will undergo transport, dilution and sedimentation processes, and some will reach the São Francisco River, overspreading the effects of the tailings release. These metals will consolidate in the bottom sediments of the Paraopeba River and may eventually be released into the water, leading to biotic accumulation and the possibility of immediate and long-term effects, such as mortality or decreases in reproduction. Particularly in relation to rare earth metals, many of their toxicological effects are unknown. In addition, due to the different uses of the water from the Paraopeba River, there is a risk of human contamination. Therefore, the present study provides the first insight into the water and sediment quality of the Paraopeba River and provides evidence for the influence of tailings as a source of metals at the sampling sites along the Paraopeba River. This initial evaluation (only five days after the rupture) demonstrated that the tailings transported along the Paraopeba River still had not reached the farther sampling sites (beyond 48 km from the dam). However, the composition of the tailings with large amounts of Fe, Al, Mn, and Ti, toxic metals such as U, Cd, Pb, As and Hg and rare earth metals such as In and Ga was toxic to different trophic levels, from primary producers such as algae to primary and secondary consumers such as microcrustaceans and fish species. Therefore, the long-term biomonitoring of the metal concentrations in abiotic matrices together with biological evaluations involving toxicological assays and field studies are necessary for the region.

## Methods

### Study area and sampling

Dam B1 was built in 1976 by the Ferteco Mining Company to receive iron ore tailings from the Córrego do Feijão and Jangada mines until it became inactive in 2016, reaching approximately 12 million m^3^ ^4^. The dam was constructed with successive layers of impoundment fills, a technique known as the “upstream” method^4,25^. The Córrego do Feijão mine is part of the Paraopeba complex in the city of Brumadinho, state of Minas Gerais, inside the Quadrilátero Ferrífero in southeast Brazil. This region is an economically active area related to the extraction of iron ore. The dam is located in the Feijão stream (also called the Ferro Carvão stream) basin, a tributary of the Paraopeba River (Supplementary Video), which in turn is a tributary of the São Francisco River, one of the most important water courses in Brazil and South America (Fig. 1). The Paraopeba River basin has a total drainage area on the order of 13,640 km², with at least 4.000 km² upstream of the dam rupture. The Feijão stream is an affluent of the right part of the Paraopeba River, its basin has an area of 32.8 km², and the average flow at its mouth is on the order of 600 L/s^6^. The main water uses of the Paraopeba River basin are electric power generation, public and industrial supply, mining and irrigation^6^. The water and sediment sampling occurred on January 30, 2019, five days after the dam rupture, at seven sampling sites: (S1) Moeda – 61.3 km upstream of the failure area, (S2) Tailings – A sample collected from the site closest to the dam failure area, (S3) Brumadinho – 5.2 km, (S4) Juatuba – 48 km, (S5) São José da Varginha – 111 km, (S6) Angueretá – 240 km, and (S7) Retiro Baixo – 302 km downstream from the dam (Fig. 1, Supplementary Table S2). At each sampling site, the physical-chemical parameters pH, electrical conductivity, dissolved oxygen and turbidity were measured. Superficial water samples were collected, fractionated and cooled for transport and analysis. One portion was reserved for the biological tests; another portion was acidified to pH 2.0 with nitric acid Suprapur (MERCK) for total metal analysis. Another subsample was filtered in 0.45 μm preweighed glass fiber filters and acidified to pH 2 for the dissolved fraction metal analysis. The last aliquot was filtered through a 0.70 μm dried preweighed glass fiber filter to obtain the suspended particulate material (SPM) through gravimetry, and the results are expressed in mg/L. The surface sediments were collected in plastic bags and kept under refrigeration at −4 °C until analysis. One sediment portion was used for granulometric characterization using a laser diffraction particle size analyzer (Shimadzu SALD-3101); another was freeze-dried, fractionated (<2 mm), and homogenized with grail and pistil for subsequent metal determinations; and the last portion was used in biological assays.

### Dissolved organic carbon (DOC) and total dissolved nitrogen (TDN) composition

Aliquots of filtered water were acidified with 2 N HCl and purged with ultrapure synthetic air. DOC and TDN were determined by high-temperature catalytic oxidation (680 °C) with an infrared dispersive detector in the Shimadzu TOC-VCPH apparatus. The values were expressed in mg/L, and the coefficient of variation was less than 5%.

### Metal determination in water and sediments

The metal determinations in water (dissolved + particulate) were performed according to USEPA method 3015 A with a microwave oven (Mars 5 Xpress-CEM Corporation) (22.5 mL water + 2.5 mL HNO_3_)^26^. The metal determinations in sediments were performed according to USEPA method 3050B by dissolving an aliquot of 0.5 g (dry weight) in a solution of HNO_3_ 65% (9 mL) + HCl 37% (4 mL) in a microwave oven (Mars 5 Xpress CEM-Corporation)^27^. The obtained results were compared to the TEL (threshold effects levels) and PEL (probable effects levels) from sediment quality guidelines established by the National Oceanic and Atmospheric Administration (NOAA). Metal determinations (Ag, Al, As, Ba, Be, Bi, Ca, Cd, Ce, Co, Cr, Cu, Dy, Er, Eu, Fe, Ga, Gd, Ho, In, La, Li, Lu, Mg, Mn, Mo, Na, Nd, Ni, P, Pb, Pr, Rb, Rh, S, Sb, Sc, Se, Sm, Sn, Tb, Th, Ti, Tl, Tm, U, V, Zn, Y, and Yb) were performed with inductively coupled plasma optical emission spectrometry (ICP-OES Varian - 720 ES), while Hg determinations were performed in a Hg Quick Trace M-750 CETAC-VARIAN analyzer. The quantification and the detection limits of the method are presented in Supplementary Table S3. The multi-element analysis was based on the tailings characteristics of the Brazilian Geological Service^14^.

### Enrichment factor (EF)

The enrichment factor is frequently used in geochemical studies to determine the degree of anthropogenic metal pollution:

$${\rm{EF}}=\frac{\left(\frac{{\rm{Metal}}}{{\rm{Ti}}}\right){\rm{sample}}}{\left(\frac{{\rm{Metal}}}{{\rm{Ti}}}\right){\rm{background}}}$$

Titanium was used as the normalizing element because of its conservative nature and low mobility, which reduce the effects of grain size and mineralogy. Background values were considered as the median of each element from the Brazilian Geological Service monitoring during 2009 and 2011^20^. According to Sakan *et al*. (2009), the EF values are interpreted as follows: EF < 1 indicates no enrichment; <3 is minor enrichment; 3–5 is moderate enrichment; 5–10 is moderately severe enrichment; 10–25 is severe enrichment; 25–50 is very severe enrichment; and >50 is extremely severe enrichment.

### Algal toxicological assay (first trophic level)

The starter cultures of *Raphidocelis subcapitata* were prepared by inoculating a loop of algal cells (from an agar plate) in 100 mL LC Oligo medium in Erlenmeyer flasks to obtain exponential cultures. The growth inhibition test was performed according to ABNT NBR 12648^28^. For the control (only with LC Oligo medium) and tested waters, three replicates were prepared in sterile flasks. A cell concentration of 1×10^4^ cells/mL was added to each replicate. The solutions were maintained under continuous agitation with white fluorescent light at 23 °C and 27 °C for 96 h. At the end of the test, three aliquots from the control and treated samples were counted under an optical microscope with a Neubauer chamber under a 100x objective.

### Daphnia similis toxicological assay (second trophic level)

The acute toxicity tests were performed according to the ABNT NBR 12713^29^, where four replicates with five neonates 6 to 24 h old were exposed to 10 mL of water samples for 48 h under static conditions at 20 ± 2 °C in the dark. The control samples were maintained only with MS medium. After exposure, immobilized organisms were counted. Tests were considered acceptable if *D. similis* immobility in the negative controls did not exceed 10%.

### Danio rerio toxicological assay (third trophic level)

This study was performed in accordance with the ethics guidelines and the experimental methods were approved by the Comissão de Ética no Uso de Animais do Campus de Alegre da Universidade Federal do Espírito Santo (CEUA-ALEGRE) with the protocol number 26/2016 and 12/2019. The acute toxicity tests were performed according to the ABNT NBR 15088^30^. Ten adult organisms (total length: 2.0 ± 1.0 cm) were exposed to the water samples for 96 h as a semi-static test, with renewal of the test solution every 48 h. The observed effect was the lethality in comparison to the control under the same conditions. The test was repeated twice as experimental replicates. *D. rerio* exemplars were also exposed to sediment samples solubilized in a proportion of 1/16 in dechlorinated water, based on the indications of ABNT NBR 10006/2004^31^ to obtain solutes from solid residues. However, the original dilution ratio of 1/4 was not followed to avoid immediate fish mortality and to evaluate a later moment after the initial impact.

### Metal accumulation in fish

An aliquot of 0.5 g (wet weight) obtained by a compost sample (5 fish of the same treatment) was solubilized in 65% HNO_3_ at 150 °C. The metal determinations (Al, Fe and Mn) were performed in resuspended 0.5% v/v HNO_3_ extracts with inductively coupled plasma optical emission spectrometry (ICP - OES Varian - 720 ES). The quantification and the detection limits of the method are presented in Supplementary Table S3.

### Statistics

The data were transformed to a z-scale (standard scale in statistics where the standard deviation is 1 unit and the mean is zero) to make the variables comparable. Pearson’s correlation was conducted to identify metal associations in each matrix, regardless of their concentrations at each sampling campaign, using the STATISTICA software, version 8.0. This analysis was conducted excluding variables with missing data. The groups were defined with pairwise variables that presented correlation coefficients greater than 0.90. The variables with missing data (with at least 3 observations) were correlated with others in the clusters when the correlation coefficients were greater than 0.90. The tendencies of metal concentrations were plotted according to the associations verified in the clusters using local polynomial regression fitting with the R software (loess, base package, R Core Team, 2019), and the shading in the plots identifies the standard error of the regression model.

The differences between toxicological tests and negative controls were determined with the R software (R Core Team, 2019). The comparisons were conducted using the nonparametric Kruskal–Wallis test (free distribution) (kruskal.test, base package, R Core Team, 2019), followed by Dunn’s multiple comparison test (dunn.test, FSA package, Ogle, D. H., Wheeler, P. and Dinno A., 2018) assuming a 95% confidence level.

## Supplementary information

Supplementary information.

Supplementary information2.

## Data Availability

The authors confirm that the data supporting the findings of this study are available within the article [and/or] its supplementary materials.

## References

[1] IBAMA. Laudo Técnico Preliminar - Impactos ambientais decorrentes do desastre envolvendo o rompimento da barragem de Fundão, em Mariana, Minas Gerais, http://www.ibama.gov.br/phocadownload/barragemdefundao/laudos/laudo_tecnico_preliminar_Ibama.pdf (2015).

[2] Carmo FF (2017). Fundão tailings dam failures: the environment tragedy of the largest technological disaster of Brazilian mining in global context. Perspect. Ecol. Conserv..

[3] Porsani J, Jesus F, Stangari M (2019). GPR survey on an iron mining area after the collapse of the tailings dam I at the Córrego do Feijão mine in Brumadinho-MG, Brazil. Remote Sens..

[4] VALE. Form 20-F: Annual repport from Securities and Exchange Commission, http://www.vale.com/EN/investors/information-market/annual-reports/20f/20FDocs/Vale_20-F%20FY2018%20-%20final_i.pdf (2019).

[5] ANM. Cadastro Nacional de Barragens – 2016, http://www.anm.gov.br/assuntos/barragens/arquivos-barragens/CADASTRO%20NACIONAL%20DE%20BARRAGENS_2016%20_FINAL%2006-01-2017.pdf/view (2017).

[6] CPRM. Monitoramento especial da bacia do Rio Paraopeba - Relatório 01: Monitoramento Hidrológico e Sedimentométrico, http://www.cprm.gov.br/sace/conteudo/paraopeba/RT_01_2019_PARAOPEBA.pdf (2019).

[7] Hatje V (2017). The environmental impacts of one of the largest tailing dam failures worldwide. Sci. Rep..

[8] Quadra G (2018). Far-reaching cytogenotoxic effects of mine waste from the Fundão dam disaster in Brazil. Chemosphere.

[9] Segura FR (2016). Potential risks of the residue from Samarco’s mine dam burst (Bento Rodrigues, Brazil). Environ. Pollut..

[10] Pandey L (2019). Towards a multi-bioassay-based index for toxicity assessment of fluvial waters. Environ. Monit. Assess..

[11] Ekelund, N.G.A. & Häder, D.P. *Bioassays* (eds Donat- P. Häder & Gilmar S. Erzinger) 419–437 (Elsevier (2018).

[12] Almeida CA (2018). Characterization and evaluation of sorption potential of the iron mine waste after Samarco dam disaster in Doce River basin – Brazil. Chemosphere.

[13] Queiroz HM (2018). The Samarco mine tailing disaster: A possible time-bomb for heavy metals contamination?. Sci. Total Environ..

[14] CPRM. Monitoramento especial da bacia do Rio Paraopeba - Relatório 03: Monitoramento Geoquímico, http://www.cprm.gov.br/sace/conteudo/paraopeba/RT_03_2019_PARAOPEBA.pdf (2019).

[15] Vicq RFC, Matschullat J, Leite M, Nalini H, Mendonça FPC (2015). Iron Quadrangle stream sediments, Brazil: Geochemical maps and reference values. Environ. Earth Sci..

[16] CONAMA. Dispõe sobre a classificação dos corpos de água e diretrizes ambientais para o seu enquadramento, bem como estabelece as condições e padrões de lançamento de efluentes, e dá outras providências - 357/2005, http://www2.mma.gov.br/port/conama/legiabre.cfm?codlegi=459 (2005).

[17] CPRM. Monitoramento especial da bacia do Rio Doce: Relatório I - Dezembro/2015, http://www.cprm.gov.br/publique/media/hidrologia/eventos_criticos/riodoce_relatorio1.pdf (2015).

[18] IGAM. Informativo hidrometeorológico de acompanhamento do deslocamento da pluma no percurso do Rio Paraopeba - N° 02/2019, http://www.meioambiente.mg.gov.br/images/stories/2019/DESASTRE_BARRAGEM_B1/informativos_hidrometeorol%C3%B3gicos/20190130_Informativo_Paraopeba_N2.pdf (2019).

[19] FEAM. Condições iniciais de avanço da lama oriunda da barragem B1 da mina Córrego do Feijão, http://www.meioambiente.mg.gov.br/images/stories/2019/DESASTRE_BARRAGEM_B1/avanco_lama/MAPA_CONDI%C3%87%C3%95ES_DE_AVAN%C3%87O_DA_PLUMA_-_30_DE_JANEIRO_-_1700H.png (2019).

[20] CPRM. Monitoramento especial da bacia do rio Paraopeba - Relatório 02: Monitoramento Geoquímico, http://www.cprm.gov.br/sace/conteudo/paraopeba/RT_02_2019_PARAOPEBA.pdf (2019).

[21] IGAM. Informativo Especial: Avaliação da série histórica entre 2000 e 2018 - Informativo dos parâmetros de qualidade das águas nos locais monitorados ao longo do Rio Paraopeba antes do desastre na barragem B1 no complexo da Mina Córrego Feijão da Mineradora Vale/SA no município de Brumadinho – Minas Gerais, http://www.meioambiente.mg.gov.br/images/stories/2019/DESASTRE_BARRAGEM_B1/informativos_qualidade_agua/Informativo_Especial__Serie_Hist%C3%B3rica_2000_a_2018_140219.pdf (2019).

[22] IGAM. Informativo semanal da avaliação dos sedimentos do rio Paraopeba nos locais monitorados ao longo do Rio Paraopeba, após o desastre na barragem B1 no complexo da Mina Córrego Feijão da Mineradora Vale/SA no município de Brumadinho – Minas Gerais - Informativo N° 2 (2019).

[23] Thompson F (2020). Severe impacts of the Brumadinho dam failure (Minas Gerais, Brazil) on the water quality of the Paraopeba River. Sci. Total Environ..

[24] ANA, IBAMA, CPRM, IGAM, UNB & Federal, P. Nota pública interinstitucional, http://www.igam.mg.gov.br/images/stories/2019/MAT%C3%89RIAS/MAIO/Nota_informativa_interinstitucional_Expedi%C3%A7%C3%A3o_Radiometria_1.pdf (2019).

[25] ICOLD. Tailings Dams - Risk of dangerous occurrences, lessons learnt from practical experiences - Bulletin 121, https://ussdams.wildapricot.org/resources/Documents/ICOLD%202001%20Bulletin%20121.pdf (2001).

[26] EPA, U.S. Method 3015A (SW-846) (2007). Microwave Assisted Acid Digestion of Aqueous Samples and Extracts. Revision.

[27] EPA, U.S. Method 3050B (1996). Acid Digestion of Sediments, Sludges, and Soils. Revision.

[28] ABNT. ABNT NBR 12648:2018 - Ecotoxicologia aquática - Toxicidade crônica - Método de ensaio com algas (Chlorophyceae). 27 (2018).

[29] ABNT. ABNT NBR 12713:2016 - Ecotoxicologia aquática - Toxicidade aguda - Método de ensaio com Daphnia spp (Crustacea, Cladocera) 27 (2016).

[30] ABNT. ABNT NBR 15088:2016 - Ecotoxicologia aquática - Toxicidade aguda - Método de ensaio com peixes (Cyprinidae) 25 (2016).

[31] ABNT. ABNT NBR 10006:2004 - Procedimento para obtenção de extrato solubilizado de resíduos sólidos. 3 (2004).

[32] IBGE. Bases Cartográficas, https://mapas.ibge.gov.br/bases-e-referenciais.html (2019).

