# Supplementary material for: Metal concentrations and biological effects from one of the largest mining disasters in the world (Brumadinho, Minas Gerais, Brazil)
Source: Sci Rep. 2020 Apr 3;10:5936. doi: 10.1038/s41598-020-62700-w (PMC7125165; doi:10.1038/s41598-020-62700-w)
Supplement: Supplementary file 1 — Supplementary information. [file 41598_2020_62700_MOESM1_ESM.pdf]

Supplementary Material

Manuscript: Metal concentrations and biological effects from one of the largest mining disasters in the world (Brumadinho, Minas Gerais, Brazil)

Authors: Cristiane dos Santos Vergilio, Diego Lacerda, Braulio Cherene Vaz de Oliveira, Echily Sartori, Gabriela Munis Campos, Anna Luiza Pereira, Diego Borges de Aguiar, Tatiana da Silva Souza, Marcelo Gomes de Almeida, Fabiano Thompson, Carlos Eduardo de Rezende

Figure S1. a) Visual aspect water collected at Brumadinho sampling site (S3 – 5.2 km of the Dam rupture area). b) The road complete filled with the mud.

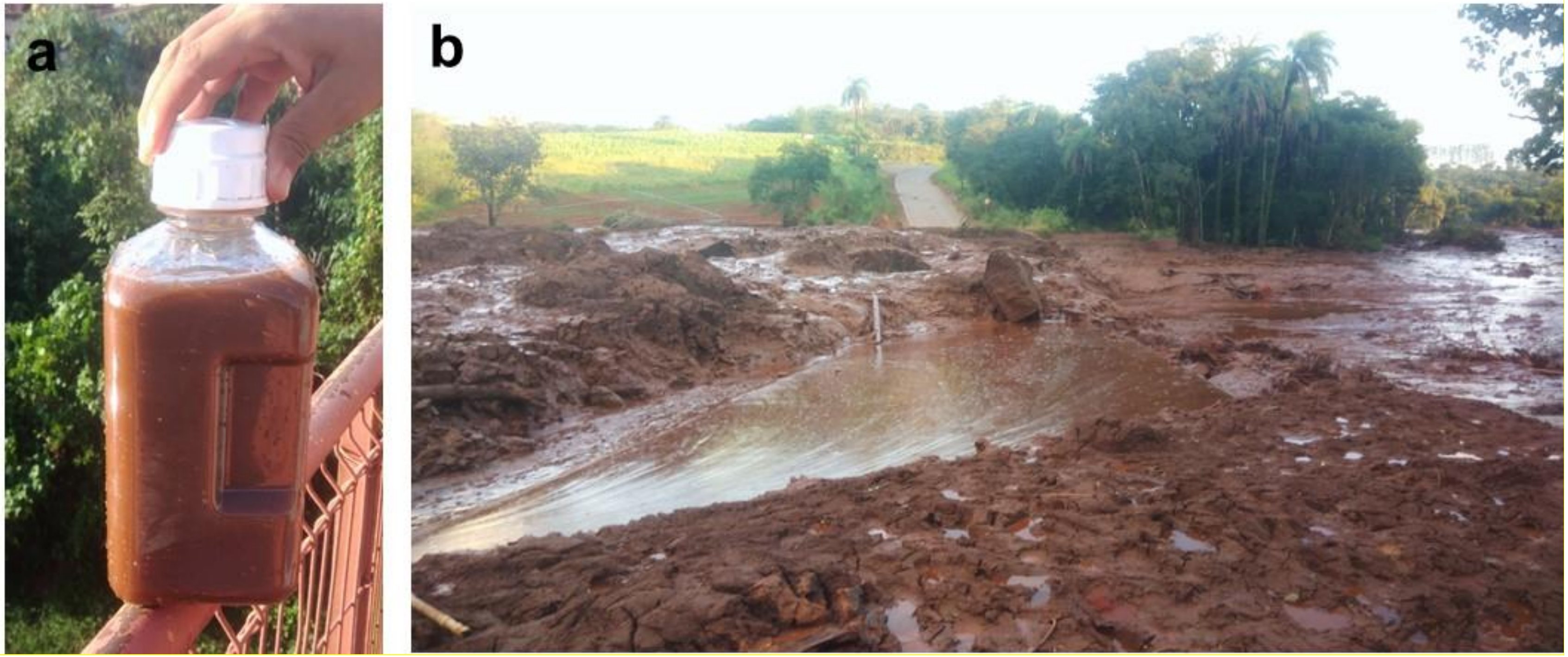

Table S2. Geospatial localization (latitude and longitude), distance of the dam, physical and chemical parameters of the sampling sites after Brumadinho dam rupture. SPM= Superficial Particulate Material, DOC = Dissolved Organic Carbon, TDN = Total Dissolved Nitrogen, DO = Dissolved Oxygen.

				Water								Sediment		
Damming distance (km)	Sites	Latitude	Longitude	SPM (mg/L)	DOC (mg/L)	TDN (mg/L)	Turbidity (NTU)	Conductivity (µs/cm)	pH	Temperature	DO (mg/L)	Sand (%)	Silt (%)	Clay (%)
-61.3	Moeda	20°20'40"S	44°02'25"W	18.36	1.99	1	6	176	7.55	27.8	4.3	100	0	0
0	Tailing	20°09'14"S	44°09'34"W	-	-	-	-	-	-	-	-	30	34	36
5.2	Brumadinho	20°08'27"S	44°12'03"W	516.14	1.34	0.69	3000	273	7.36	28	3.92	50	27	23
48	Juatuba	19°56'56"S	44°18'18"W	8.44	1.94	1.47	6.1	233	7.98	27.7	4.63	38	40	22
111	São José da Varginha	19°40'13"S	44°28'51"W	18.8	1.67	1.43	5.2	229	7.25	30	6.15	53	30	16
240	Anguereta	19°10'25"S	44°42'05"W	8.32	2.21	1.18	3.6	251	7.45	29.1	5.52	42	36	22
302	Retiro Baixo	18°54'09"S	44°47'02"W	4.45	6.16	0.69	1.7	177	7.55	30.4	5.32	36	38	26

Table S3. Method's detection and quantification limits of the metals analyzed in water (mg/L), sediment and fish (µg/g) and samples validation of the fish analytical procedure using DORM 3 standard reference material. DL = detection limit; QL = quantification limit. *Hg values are expressed in ng/L (water) and ng/g (sediment).

		Ag	Al	As	Ba	Be	Bi	Ca	Cd	Ce	Co	Cr	Cu	Dy	Er	Eu	Fe	Ga	Gd
Water	DL	0.006	0.0009	0.006	0.0002	0.00006	0.002	0.009	0.0001	0.0016	0.0005	0.0003	0.0006	0.0002	0.0003	0.0001	0.0006	0.003	0.0004
	QL	0.02	0.003	0.021	0.0007	0.0002	0.008	0.0297	0.0005	0.0053	0.0017	0.001	0.002	0.0006	0.0009	0.0004	0.0021	0.01	0.0013
Sediment	DL	0.5	0.7	0.9	0.3	0.09	1.62	0.9	0.1	0.78	0.5	0.4	0.4	0.43	0.4	0.44	0.7	0.66	0.49
	QL	1.65	2.31	2.97	0.99	0.28	5.35	2.97	0.33	2.56	1.65	1.32	1.32	1.41	1.33	1.46	2.31	2.18	1.61
Fish	DL	-	0.14	-	-	-	-	-	-	-	-	-	-	-	-	-	0.14	-	-
	QL	-	0.462	-	-	-	-	-	-	-	-	-	-	-	-	-	0.462	-	-
	Recovery (%)	-	100	-	-	-	-	-	-	-	-	-	-	-	-	-	100	-	-

		Hg	Ho	In	K	La	Li	Lu	Mg	Mn	Mo	Na	Nd	Ni	P	Pb	Pr	Rb	Rh
Water	DL	0.06*	0.0002	0.002	0.009	0.0004	0.0002	0.0002	0.008	0.0004	0.001	0.009	0.0009	0.0005	0.008	0.002	0.002	0.004	0.0009
	QL	0.2*	0.0008	0.006	0.0297	0.0013	0.0006	0.0008	0.018	0.0012	0.005	0.023	0.0029	0.001	0.0264	0.007	0.006	0.013	0.003
Sediment	DL	0.083*	0.42	1.41	0.9	0.46	0.08	0.47	0.8	0.3	1.25	0.91	0.53	0.4	0.8	0.4	0.59	0.67	0.66
	QL	0.25*	1.4	4.65	2.97	1.52	0.27	1.56	1.8	0.99	4.12	2.26	1.74	1.32	2.64	1.32	1.93	2.23	2.18
Fish	DL	-	-	-	-	-	-	-	-	0.06	-	-	-	-	-	-	-	-	-
	QL	-	-	-	-	-	-	-	-	0.198	-	-	-	-	-	-	-	-	-
	Recovery (%)	-	-	-	-	-	-	-	-	84	-	-	-	-	-	-	-	-	-

		S	Sb	Sc	Se	Sm	Sn	Sr	Tb	Th	Ti	Tl	Tm	U	V	Y	Yb	Zn
Water	DL	0.15	0.004	0.0002	0.01	0.002	0.004	0.001	0.0006	0.002	0.0003	0.009	0.001	0.009	0.004	0.0002	0.0002	0.002
	QL	0.495	0.013	0.0008	0.04	0.007	0.013	0.003	0.0019	0.005	0.001	0.029	0.004	0.029	0.0132	0.0008	0.0006	0.006
Sediment	DL	15	0.53	0.55	0.9	0.47	0.4	0.10	0.51	1.04	0.3	0.73	0.49	0.91	0.4	0.46	0.46	0.3
	QL	49.5	1.73	1.81	2.97	1.57	1.32	0.33	1.7	3.45	0.99	2.4	1.63	3	1.32	1.53	1.51	0.99
Fish	DL	-	-	-	-	-	-	-	-	-	-	-	-	-	-	-	-	-
	QL	-	-	-	-	-	-	-	-	-	-	-	-	-	-	-	-	-
	Recovery (%)	-	-	-	-	-	-	-	-	-	-	-	-	-	-	-	-	-

Table S4. Metal concentrations in the water (total and dissolved) of the sampling sites after the Brumadinho Dam rupture. CONAMA values with * are set from the dissolved fraction.

Sampling site	Compartiment	Damming distance (km)	Ag (mg/L)	Al (mg/L)	As (mg/L)	Ba (mg/L)	Be (mg/L)	Bi (mg/L)	Ca (mg/L)	Cd (mg/L)	Ce (mg/L)	Co (mg/L)	Cr (mg/L)	Cu (mg/L)
1 - Moeda	Water (Total)	-61.3	<0.0060	0.76	<0.0062	0.020	<0.0001	<0.0024	5.33	<0.0001	0.005	0.0047	0.005	0.015
3 - Brumadinho	Water (Total)	5.2	<0.0060	10.02	<0.0062	0.121	<0.0001	<0.0024	5.30	0.007	0.010	0.0115	0.010	0.079
4 - Juatuba	Water (Total)	48	<0.0060	0.58	<0.0062	0.011	<0.0001	<0.0024	6.39	<0.0001	<0.0016	<0.0005	0.007	0.025
5 - São José da Varginha	Water (Total)	111	<0.0060	0.69	<0.0062	0.022	<0.0001	<0.0024	6.71	<0.0001	<0.0016	<0.0005	0.002	0.014
6 - Anguereta	Water (Total)	240	<0.0060	0.38	<0.0062	0.020	<0.0001	<0.0024	6.33	<0.0001	<0.0016	<0.0005	<0.0003	0.019
7 - Retiro Baixo	Water (Total)	302	<0.0060	0.27	<0.0062	0.020	<0.0001	<0.0024	6.35	<0.0001	<0.0016	<0.0005	<0.0003	0.021
1 - Moeda	Water (Dissolved)	-61.3	<0.0060	0.27	<0.0062	0.015	<0.0001	<0.0024	2.78	<0.0001	<0.0016	<0.0005	<0.0003	0.007
3 - Brumadinho	Water (Dissolved)	5.2	<0.0060	0.20	<0.0062	0.100	<0.0001	<0.0024	3.29	0.0008	0.0018	<0.0005	<0.0003	<0.0006
4 - Juatuba	Water (Dissolved)	48	<0.0060	0.04	<0.0062	0.007	<0.0001	<0.0024	3.60	<0.0001	<0.0016	<0.0005	<0.0003	0.007
5 - São José da Varginha	Water (Dissolved)	111	<0.0060	0.08	<0.0062	0.012	<0.0001	<0.0024	4.46	<0.0001	<0.0016	<0.0005	0.001	0.001
6 - Anguereta	Water (Dissolved)	240	<0.0060	0.03	<0.0062	0.006	<0.0001	<0.0024	4.82	<0.0001	<0.0016	<0.0005	<0.0003	<0.0006
7 - Retiro Baixo	Water (Dissolved)	302	<0.0060	0.18	<0.0062	0.009	<0.0001	<0.0024	5.42	<0.0001	<0.0016	<0.0005	<0.0003	<0.0006
	CONAMA	I	0.01	0.10*	0.01	0.70	0.04	-	-	0.001	-	0.0500	0.05	0.009*
		III	0.05	0.20*	0.033	1.00	0.10	-	-	0.010	-	0.2000	0.05	0.013*

Sampling site	Compartiment	Damming distance (km)	Dy (mg/L)	Er (mg/L)	Eu (mg/L)	Fe (mg/L)	Ga (mg/L)	Gd (mg/L)	Hg (ng/L)	Ho (mg/L)	In (mg/L)	K (mg/L)	La (mg/L)	Li (mg/L)
1 - Moeda	Water (Total)	-61.3	<0.0002	<0.0003	<0.0001	1.39	<0.0031	<0.0004	3.55	<0.0002	<0.0018	1.52	0.0041	0.0013
3 - Brumadinho	Water (Total)	5.2	<0.0002	0.001	<0.0001	48.15	<0.0031	0.009	144.26	0.0004	0.2019	1.53	0.012	0.0041
4 - Juatuba	Water (Total)	48	<0.0002	<0.0003	<0.0001	0.87	<0.0031	<0.0004	4.12	<0.0002	0.0089	2.12	<0.0004	0.0007
5 - São José da Varginha	Water (Total)	111	<0.0002	<0.0003	<0.0001	0.76	<0.0031	<0.0004	4.16	<0.0002	<0.0018	1.92	<0.0004	0.0005
6 - Anguereta	Water (Total)	240	<0.0002	<0.0003	<0.0001	0.50	<0.0031	<0.0004	2.46	<0.0002	<0.0018	1.80	<0.0004	0.0004
7 - Retiro Baixo	Water (Total)	302	<0.0002	<0.0003	<0.0001	0.28	<0.0031	<0.0004	1.51	<0.0002	<0.0018	1.92	<0.0004	0.0004
1 - Moeda	Water (Dissolved)	-61.3	<0.0002	<0.0003	<0.0001	0.38	<0.0031	<0.0004	1.88	<0.0002	<0.0018	1.28	<0.0004	0.0009
3 - Brumadinho	Water (Dissolved)	5.2	<0.0002	<0.0003	<0.0001	0.84	<0.0031	<0.0004	1.86	<0.0002	<0.0018	1.19	<0.0004	0.0013
4 - Juatuba	Water (Dissolved)	48	<0.0002	<0.0003	<0.0001	0.02	<0.0031	<0.0004	1.01	<0.0002	0.0028	1.76	<0.0004	0.0004
5 - São José da Varginha	Water (Dissolved)	111	<0.0002	<0.0003	<0.0001	0.07	<0.0031	<0.0004	1.90	<0.0002	<0.0018	1.35	<0.0004	<0.0002
6 - Anguereta	Water (Dissolved)	240	<0.0002	<0.0003	<0.0001	0.01	<0.0031	<0.0004	1.31	<0.0002	<0.0018	1.45	<0.0004	<0.0002
7 - Retiro Baixo	Water (Dissolved)	302	<0.0002	<0.0003	<0.0001	0.12	<0.0031	<0.0004	0.66	<0.0002	<0.0018	1.00	<0.0004	<0.0002
	CONAMA	I	-	-	-	0.30*	-	-	200	-	-	-	-	2.5000
		III	-	-	-	5.00*	-	-	2000	-	-	-	-	2.5000

CONAMA: Conselho Nacional do Meio Ambiente – “National Council of Environment”

Table S4. Continuation.

Sampling site	Compartiment	Damming distance (km)	Lu (mg/L)	Mg (mg/L)	Mn (mg/L)	Mo (mg/L)	Na (mg/L)	Nd (mg/L)	Ni (mg/L)	P (mg/L)	Pb (mg/L)	Pr (mg/L)	Rb (mg/L)	Rh (mg/L)
1 - Moeda	Water (Total)	-61.3	0.0004	1.88	0.066	<0.0014	6.15	0.025	0.005	<0.0029	0.003	<0.0017	0.005	<0.0009
3 - Brumadinho	Water (Total)	5.2	0.001	2.20	5.113	<0.0014	6.55	0.029	0.014	0.4554	0.020	<0.0017	0.009	<0.0009
4 - Juatuba	Water (Total)	48	<0.0002	2.04	0.182	<0.0014	8.00	0.028	0.013	<0.0029	0.003	<0.0017	0.008	<0.0009
5 - São José da Varginha	Water (Total)	111	<0.0002	1.85	0.048	<0.0014	7.24	0.019	<0.0005	<0.0029	<0.0019	<0.0017	0.009	<0.0009
6 - Anguereta	Water (Total)	240	<0.0002	1.96	0.019	<0.0014	7.56	0.026	<0.0005	<0.0029	<0.0019	<0.0017	0.010	<0.0009
7 - Retiro Baixo	Water (Total)	302	<0.0002	1.82	0.007	<0.0014	5.39	0.027	<0.0005	0.0049	0.003	<0.0017	0.011	<0.0009
1 - Moeda	Water (Dissolved)	-61	<0.0002	1.34	0.005	<0.0014	3.26	0.018	0.0024	<0.0029	0.002	<0.0017	<0.0039	<0.0009
3 - Brumadinho	Water (Dissolved)	5	0.0003	1.79	0.039	<0.0014	4.28	0.018	<0.0003	<0.0029	0.005	<0.0017	<0.0039	<0.0009
4 - Juatuba	Water (Dissolved)	48	<0.0002	1.69	0.015	<0.0014	6.00	0.017	0.0061	<0.0029	0.003	<0.0017	<0.0039	<0.0009
5 - São José da Varginha	Water (Dissolved)	111	<0.0002	1.24	0.002	<0.0014	5.10	0.013	<0.0005	<0.0029	<0.0019	<0.0017	<0.0039	<0.0009
6 - Anguereta	Water (Dissolved)	240	<0.0002	1.27	<0.0004	<0.0014	4.58	0.019	<0.0005	<0.0029	<0.0019	<0.0017	<0.0039	<0.0009
7 - Retiro Baixo	Water (Dissolved)	302	<0.0002	1.40	<0.0004	<0.0014	3.88	0.016	<0.0005	0.0031	<0.0019	<0.0017	<0.0039	<0.0009
	CONAMA	I	-	-	0.10	-	-	-	0.025	0.1000	0.010	-	-	-
		III	-	-	0.50	-	-	-	0.025	0.1500	0.033	-	-	-

Sampling site	Compartiment	Damming distance (km)	S (mg/L)	Sb (mg/L)	Sc (mg/L)	Se (mg/L)	Sm (mg/L)	Sn (mg/L)	Sr (mg/L)	Tb (mg/L)	Th (mg/L)	Ti (mg/L)	Tl (mg/L)	Tm (mg/L)
1 - Moeda	Water (Total)	-61.3	1.60	<0.0041	0.0015	<0.0132	<0.0021	0.0537	0.050	<0.0006	<0.0016	0.013	<0.0087	<0.0011
3 - Brumadinho	Water (Total)	5.2	1.33	<0.0041	0.003	<0.0132	<0.0021	<0.0040	0.053	<0.0006	<0.0016	0.124	<0.0087	<0.0011
4 - Juatuba	Water (Total)	48	2.58	<0.0041	<0.0002	<0.0132	<0.0021	<0.0040	0.035	<0.0006	<0.0016	0.010	<0.0087	<0.0011
5 - São José da Varginha	Water (Total)	111	1.93	<0.0041	<0.0002	<0.0132	<0.0021	<0.0040	0.036	<0.0006	<0.0016	0.010	<0.0087	<0.0011
6 - Anguereta	Water (Total)	240	1.00	<0.0041	<0.0002	<0.0132	<0.0021	<0.0040	0.040	<0.0006	<0.0016	0.005	<0.0087	<0.0011
7 - Retiro Baixo	Water (Total)	302	0.97	<0.0041	<0.0002	<0.0132	<0.0021	<0.0040	0.040	<0.0006	<0.0016	0.004	<0.0087	<0.0011
1 - Moeda	Water (Dissolved)	-61.3	1.19	<0.0041	0.0005	<0.0132	<0.0021	<0.0040	0.036	<0.0006	<0.0016	0.001	<0.0087	<0.0011
3 - Brumadinho	Water (Dissolved)	5.2	1.00	<0.0041	0.0003	<0.0132	<0.0021	<0.0040	0.045	<0.0006	<0.0016	0.005	<0.0087	<0.0011
4 - Juatuba	Water (Dissolved)	48	1.45	<0.0041	<0.0002	<0.0132	<0.0021	<0.0040	0.030	<0.0006	<0.0016	0.008	<0.0087	<0.0011
5 - São José da Varginha	Water (Dissolved)	111	1.29	<0.0041	<0.0002	<0.0132	<0.0021	<0.0040	0.023	<0.0006	<0.0016	0.001	<0.0087	<0.0011
6 - Anguereta	Water (Dissolved)	240	0.59	<0.0041	<0.0002	<0.0132	<0.0021	<0.0040	0.016	<0.0006	<0.0016	0.004	<0.0087	<0.0011
7 - Retiro Baixo	Water (Dissolved)	302	0.45	<0.0041	<0.0002	<0.0132	<0.0021	<0.0040	0.004	<0.0006	<0.0016	0.003	<0.0087	<0.0011
	CONAMA	I	-	0.0050	-	0.01	-	-	-	-	-	-	-	-
		III	-	-	-	0.05	-	-	-	-	-	-	-	-

Table S4. Continuation.

Sampling site	Compartiment	Damming distance (km)	U (mg/L)	V (mg/L)	Y (mg/L)	Yb (mg/L)	Zn (mg/L)
1 - Moeda	Water (Total)	-61.3	0.06	<0.0006	0.007	<0.0002	0.016
3 - Brumadinho	Water (Total)	5.2	0.14	0.007	0.017	<0.0002	0.042
4 - Juatuba	Water (Total)	48	<0.0088	<0.0006	0.000	<0.0002	0.054
5 - São José da Varginha	Water (Total)	111	<0.0088	<0.0006	<0.0002	<0.0002	0.005
6 - Anguereta	Water (Total)	240	<0.0088	<0.0006	<0.0002	<0.0002	<0.0018
7 - Retiro Baixo	Water (Total)	302	<0.0088	<0.0006	<0.0002	<0.0002	<0.0018
1 - Moeda	Water (Dissolved)	-61	<0.0088	<0.0006	0.0005	<0.0002	0.004
3 - Brumadinho	Water (Dissolved)	5	<0.0088	<0.0006	<0.0002	<0.0002	0.029
4 - Juatuba	Water (Dissolved)	48	<0.0088	<0.0006	<0.0002	<0.0002	0.023
5 - São José da Varginha	Water (Dissolved)	111	<0.0088	<0.0006	<0.0002	<0.0002	0.003
6 - Anguereta	Water (Dissolved)	240	<0.0088	<0.0006	<0.0002	<0.0002	<0.0018
7 - Retiro Baixo	Water (Dissolved)	302	<0.0088	<0.0006	<0.0002	<0.0002	<0.0018
	CONAMA	I	0.02	0.10	-	-	0.18
		III	0.02	0.10	-	-	5.00

Table S5. Metal concentrations in the sediment of the sampling sites after the Brumadinho Dam rupture.

Sampling site	Damming distance (km)	Ag (µg/g)	Al (mg/g)	As (µg/g)	Ba (µg/g)	Be (µg/g)	Bi (µg/g)	Ca (µg/g)	Cd (µg/g)	Ce (µg/g)	Co (µg/g)	Cr (µg/g)	Cu (µg/g)
1 - Moeda	-61.3	<0.50	8.9	10.23	135.3	<0.09	2.82	292	2.62	26.88	28.21	44.4	8.94
2 - Tailing	0	1.31	10.8	4.69	76.6	0.49	15.20	161	30.94	15.05	10.43	16.4	36.03
3 - Brumadinho	5.2	2.31	21.7	2.43	121.8	0.91	13.24	311	32.57	9.72	14.09	28.0	66.42
4 - Juatuba	48	0.81	75.8	3.68	235.9	0.88	<1.62	843	7.62	34.86	10.09	50.2	24.00
5 - São José da Varginha	111	<0.50	9.9	<0.90	138.0	<0.09	2.05	246	2.32	10.11	8.74	10.0	5.92
6 - Anguereta	240	0.95	47.9	11.86	134.8	1.22	<1.62	288	8.99	8.00	14.76	92.3	36.98
7 - Retiro Baixo	302	0.84	37.2	14.52	158.4	1.31	<1.62	159	7.39	45.51	13.79	156.1	38.24
	TEL	-	-	5.90	-	-	-	-	0.60	-	-	37.3	35.7
	PEL	-	-	17.0	-	-	-	-	3.53	-	-	90	197

Sampling site	Damming distance (km)	Dy (µg/g)	Er (µg/g)	Eu (µg/g)	Fe (mg/g)	Ga (µg/g)	Gd (µg/g)	Hg (ng/g)	Ho (µg/g)	In (µg/g)	K (mg/g)	La (µg/g)	Li (µg/g)
1 - Moeda	-61.3	0.76	<0.40	<0.44	16.1	1.08	1.77	32.0	<0.42	56.3	2625	2.80	7.42
2 - Tailing	0	5.13	0.70	1.61	264.9	92.34	50.61	101.3	<0.42	210.2	354	22.20	8.67
3 - Brumadinho	5.2	7.36	0.98	1.18	279.6	113.85	48.49	184.5	<0.42	402.6	961	13.82	17.32
4 - Juatuba	48	1.47	0.96	1.33	71.9	51.27	12.17	51.5	<0.42	26.1	13723	16.69	24.65
5 - São José da Varginha	111	0.86	<0.40	<0.44	4.9	<0.66	0.53	14.0	<0.42	11.1	5450	1.82	3.57
6 - Anguereta	240	3.72	1.08	1.13	71.3	59.62	12.28	63.0	<0.42	57.3	13040	4.41	28.47
7 - Retiro Baixo	302	0.98	1.74	1.44	53.3	48.92	10.67	49.8	<0.42	44.6	20412	10.75	25.62
	TEL	-	-	-	-	-	-	174	-	-	-	-	-
	PEL	-	-	-	-	-	-	486	-	-	-	-	-

Sampling site	Damming distance (km)	Lu (µg/g)	Mg (µg/g)	Mn (mg/g)	Mo (µg/g)	Na (µg/g)	Nd (µg/g)	Ni (µg/g)	P (µg/g)	Pb (µg/g)	Pr (µg/g)	Rb (µg/g)	Rh (µg/g)
1 - Moeda	-61.3	0.49	211	0.99	<1.25	874	9.99	22.03	50	3.03	1.02	15.65	<0.66
2 - Tailing	0	6.33	236	4.78	<1.25	232	14.19	17.20	1031	14.65	39.48	2.26	<0.66
3 - Brumadinho	5.2	6.09	464	10.51	<1.25	139	10.28	24.16	1347	18.38	37.65	2.78	<0.66
4 - Juatuba	48	1.60	970	0.99	<1.25	2428	22.85	18.56	506	14.87	9.99	72.31	<0.66
5 - São José da Varginha	111	<0.47	113	0.24	<1.25	1567	9.46	9.58	26	<0.40	<0.59	31.90	<0.66
6 - Anguereta	240	1.66	315	2.59	<1.25	961	8.70	32.84	576	10.01	9.49	22.97	<0.66
7 - Retiro Baixo	302	1.45	960	0.53	<1.25	460	15.54	32.18	346	12.90	9.28	56.60	<0.66
	TEL	-	-	-	-	-	-	18	-	35.8	-	-	-
	PEL	-	-	-	-	-	-	36	-	91.3	-	-	-

Table S5. Continuation.

Sampling site	Damming distance (km)	S (µg/g)	Sb (µg/g)	Sc (µg/g)	Se (µg/g)	Sm (µg/g)	Sn (µg/g)	Sr (µg/g)	Tb (µg/g)	Th (µg/g)	Ti (mg/g)	Tl (µg/g)	Tm (µg/g)
1 - Moeda	-61.3	280	<0.53	1.63	<0.90	<0.47	20.6	12.6	2.48	<1.04	0.40	<0.73	<0.49
2 - Tailing	0	241	0.69	2.41	<0.90	<0.47	547.4	15.0	42.18	<1.04	0.43	<0.73	<0.49
3 - Brumadinho	5.2	266	1.65	2.52	<0.90	<0.47	619.8	26.8	43.87	<1.04	0.59	<0.73	<0.49
4 - Juatuba	48	417	<0.53	7.10	<0.90	<0.47	146.3	19.3	7.89	<1.04	2.93	<0.73	<0.49
5 - São José da Varginha	111	189	2.94	0.69	<0.90	<0.47	0.7	16.0	1.06	<1.04	0.21	<0.73	<0.49
6 - Anguereta	240	394	<0.53	3.56	<0.90	<0.47	161.2	<0.10	8.29	<1.04	3.81	<0.73	<0.49
7 - Retiro Baixo	302	339	<0.53	5.72	<0.90	<0.47	117.7	<0.10	5.73	<1.04	3.46	<0.73	<0.49
	TEL	-	-	-	-	-	-	-	-	-	-	-	-
	PEL	-	-	-	-	-	-	-	-	-	-	-	-

Sampling site	Damming distance (km)	U (µg/g)	V (µg/g)	Y (µg/g)	Yb (µg/g)	Zn (µg/g)
1 - Moeda	-61.3	57.1	23.54	1.58	<0.46	11.86
2 - Tailing	0	1457.4	27.81	13.96	1.30	68.50
3 - Brumadinho	5.2	1674.1	36.08	11.42	1.34	89.51
4 - Juatuba	48	390.3	74.37	6.95	0.62	79.38
5 - São José da Varginha	111	21.5	7.67	1.17	<0.46	6.16
6 - Anguereta	240	421.0	126.11	0.67	<0.46	76.04
7 – Retiro Baixo	302	318.3	125.08	5.08	0.59	48.98
	TEL	-	-	-	-	123
	PEL	-	-	-	-	315

Table S6. Metal concentrations in the *D. rerio* muscle of the sampling sites after the Brumadinho Dam rupture. ± standard deviation.

Compartiment	Damming distance (km)	Sites	Al	Fe	Mn
Water	-61.3	Moeda	10.04 ± 5.43	17.99 ± 8.11	6.3 ± 4.88
	5.2	Brumadinho	16.29 ± 11.55	18.84 ± 4.44	6.16 ± 5.67
	48	Juatuba	13.96 ± 1.66	17.13 ± 2.04	2.47 ± 0.27
	111	São José da Varginha	19.41 ± 14.33	19.04 ± 2.44	1.87 ± 0.2
	240	Anguereta	10.37 ± 5.01	19.68 ± 8.1	1.77 ± 0.11
	302	Retiro Baixo	8.15 ± 3.52	10.91 ± 1.13	1.57 ± 0.05
		Mean	13.04 ± 4.29	17.27 ± 3.24	3.36 ± 2.25
Sediment	-61.3	Moeda	4.99 ± 0.81	12.93 ± 9.15	1.91 ± 0.15
	5.2	Brumadinho	7.75 ± 5.81	37.71 ± 9.32	5.93 ± 2.01
	48	Juatuba	22.49 ± 2.03	16.88 ± 4.33	2.69 ± 0.58
	111	São José da Varginha	2.99	5.35 ± 0.32	2.21 ± 0.05
	240	Angueretá	12.83 ± 7.99	11.49 ± 0.21	2.61 ± 0.32
	302	Retiro Baixo	11.5 ± 0.72	8.44 ± 0.34	1.35 ± 0.02
		Mean	10.43 ± 6.99	15.47 ± 11.6	2.78 ± 1.62
	control		3.33 ± 3.27	5.99 ± 3.99	1.27 ± 0.25

Video. Video with satellite images (©Maxar Technologies) obtained from Google Earth Pro (version 7.3.2.5776), before (July 21, 2018) and after the dam failure (March 14, 2019). The video was produced using the power point software (Microsoft Office Professional Plus - 2019 - Version 1808).
